# Go Ask Your Patients! PSS-QoL Reported Perception of Dryness Correlates With Lacrimal and Salivary Flow in Primary Sjögren's Syndrome

**DOI:** 10.3389/fmed.2021.660580

**Published:** 2021-04-15

**Authors:** Angelika Lackner, Philipp Bosch, Sabine Zenz, Jutta Horwath-Winter, Dieter Franz Rabensteiner, Josef Hermann, Winfried Graninger, Martin Helmut Stradner

**Affiliations:** ^1^Department of Rheumatology and Immunology, Medical University of Graz, Graz, Austria; ^2^Department of Ophtalmology, Medical University of Graz, Graz, Austria

**Keywords:** Sjögren syndrome, dryness, quality of life, dryness assessment, disease activity

## Abstract

**Introduction/Objectives:** The patient perspective is an essential outcome parameter in the quest for effective therapy in primary Sjögren's Syndrome (PSS). The EULAR Sjögren's Syndrome Patient Reported Index (ESSPRI) is recommended by EULAR to quantify patient's symptom burden and has been used in several clinical trials. Surprisingly, the patient's perception of dryness quantified with ESSPRI does not correlate with objective measures of salivary or lacrimal flow. Thus, we evaluated a newly developed assessment tool-the Primary Sjögren's Syndrome Quality of Life Questionnaire (PSS-QoL)—for quantifying symptoms of dryness in comparison with the ESSPRI and objective measurements of salivary and lacrimal flow.

**Methods:** Data of patients from the PSS registry of the Medical University of Graz fulfilling the 2016 ACR/EULAR classification criteria for PSS were analyzed. The patient perspective was analyzed by PSS-QoL, ESSPRI, Xerostomia Inventory (XI) and Ocular Surface Disease Index (OSDI). Sicca signs were measured with Schirmer's test, unstimulated salivary flow test (USF) and stimulated salivary flow test (SSF). ESSDAI (EULAR Sjögren's Syndrome Disease Activity Index) and EGA (Evaluator Global Assessment, numeric rating scale from 0 to 10) were obtained. In addition, free light chains (FLC) κ and λ, rheumatoid factor (RF) IgM and IgA were determined.

**Results:** Data from 123 PSS patients were analyzed; 91.9% (*n* = 113) were female, with a mean disease duration of 6.2 (±5.3) years and mean age of 60.1 (±12.4) years. PSS-QoL-dryness revealed significant negative correlations with Schirmer's test (*r* = −0.31, *p* < 0.05) and SSF-test (*r* = −0.390, *p* < 0.01). In contrast, we found no significant correlation between ESSPRI-dryness and any objective dryness test. Lower perceived dryness was associated with higher immunological activity determined by increased levels of IgG, FLC and RF-IgA. Whereas patients with only subjective signs of dryness had lower immunological activity.

**Discussion:** Patients' perception of dryness assessed by PSS-QoL correlates with objective measurements of salivary gland function while ESSPRI-dryness did not. Based on the PSS-QoL and objective measures of dryness two distinct groups of PSS patients could be distinguished, which may have implications in daily practice and future clinical studies.

## Introduction

Primary Sjögren's Syndrome (PSS) is an autoimmune disease leading to inflammation of lacrimal and salivary glands causing dryness of the eyes and mouth. Other symptoms of dryness affect the gastrointestinal and genital tract as well as skin. A considerable proportion of patients also experience extraglandular manifestations including fatigue, musculoskeletal, gastrointestinal and/or neurological symptoms (1). Several factors contribute to health-related quality of life (HRQL) in PSS patients (2), that serve as outcome parameters for current and novel therapeutic approaches (3). Currently, effective treatment options are lacking in PSS. Therefore, therapy is mainly symptomatic focusing on the management of sicca symptoms, pain and fatigue (4, 5). Patients consider these three main symptoms as more important than extraglandular manifestations (2). There is only a weak relationship between subjective and objective assessment methods of dryness (6–9). This issue may partly be responsible for difficulties detecting therapeutic efficacy in clinical trials. Understanding the association between objective and subjective dryness is of great importance to improve research outcomes in PSS and to optimize patient care in clinical practice. Some attempts were performed to solve this dilemma and quantify patients for clinical trials (9, 10): patients were classified by the degree of discrepancy between the objective and subjective symptom class (9) or patients were classified by the intent of their symptom burden (dependent of dryness, pain, fatigue, anxiety and depression) (10).

The EULAR Sjögren's Syndrome Patient Reported Index (ESSPRI) (11) is recommended by EULAR to quantify patient's symptom burden and has been used in several clinical trials. ESSPRI consists of three questions about overall dryness, fatigue and pain. However, the patient's perception of dryness quantified with ESSPRI weakly correlated with objective measures of salivary or lacrimal flow. This correlation is weaker in ocular compared to oral dryness (9). ESSPRI considers overall dryness perception and is not able to identify the origin of dryness or dryness-related symptoms. Therefore, a new more specific assessment tool is urgently needed to evaluate HRQL including dryness. We have previously published the Primary Sjögren's Syndrome Quality of Life questionnaire (PSS-QoL) (12). This tool is a disease-specific HRQL questionnaire, considering patients' perspective. One advantage of this tool is the assessment of dryness of all affected body parts separately and the evaluation of additional dryness-related symptoms. Thus, the relationship between subjective and objective measurements of dryness could be improved by evaluating the exact location of dryness. In addition to dryness-related quality of life, it assesses other important domains of HRQL affecting PSS-patients daily life. Here, we evaluated PSS-QoL for quantifying symptoms of dryness in comparison to the ESSPRI and objective measurements of salivary and lacrimal flow.

## Materials and Methods

Clinical data of patients from the PSS registry cohort of the Division of Rheumatology and Immunology, Medical University of Graz, Austria, were prospectively collected. Participants fulfilled the 2016 ACR/EULAR classification criteria for PSS (13). All participants gave written informed consent, and the study was approved by the institutional review board of the Medical University of Graz (30–101 ex 17/18).

### Objective Dryness Assessment

As part of the Sjögrens' registry, the following objective measurements of dryness were performed: Schirmer's test, unstimulated salivary flow test (USF), stimulated salivary flow test (SSF).

#### Ocular Dryness

For the Schirmer's test I, a sterile filter paper was inserted inside the patient's lower eye lid for 5 min and the degree of wetting was measured. Participants were asked not to use eye-drops for 2 h prior to testing. A Schirmer's test result of ≤ 5 mm/5 min was considered pathologic with an abnormal tear production (13).

In a subset of patients, further objective ophtalmologic assessments of eye-dryness were performed at the dry-eye unit of the department of Ophthalmology of the Medical University of Graz by experienced physicians (JHW, DR): fluorescein tear film break-up time (F-BUT), corneal fluorescein staining (CFS)(NEI – Score 0–15), Lissamine-green staining (van Bijsterveld) (Score from 0–9), Marx-line, expressibility and quality of meibomian gland secretion. F-BUT was determined after the application of dye (1 ml of 1% fluorescein solution) into the tear film. The patient was instructed to blink a few times and then to keep eyes open. The precorneal tear film was observed at 10-fold magnification using a slit lamp with cobalt blue illumination. By a stopwatch, the time until the break-up of the tear film was measured three times and the mean was documented (pathologic value ≤ 5 s). Subsequently, the extent of fluorescein staining of the cornea was reported using area and density according to the NEI-score (pathologic value >3) (14). The ocular surface was further evaluated by lissamine green staining. The lissamine green strips were moistened with one drop of physiological saline and dye was introduced into the lower conjunctional sac. Staining of the nasal, central and temporal third of the ocular surface was scored according to van Bijsterveld: a total score of 9 points per eye (pathologic value ≥3.5) (15). Lissamine green also stains the Marx line, which is usually located on the conjunctival side of the orifices of the meibomian glands. A score is defined for the outer, the middle and the inner third of the upper and lower lids. The scores of the three portions were summarized and defined as the total score of each lid (maximum score of 9, pathologic score >3) (16).

Expressibility of Meibomian glands and quality of secretion were assessed by applying pressure with a cotton tip to the skin of the middle of the lower and upper lid (score 0–3, pathologic score >1) (17).

#### Oral Dryness

For the unstimulated salivary flow test (USF), patients spit saliva into a graduated test tube every minute for a total of 5 min. USF was considered as abnormal if saliva quantity was ≤ 0.1 ml per minute (13).

After the USF, a stimulated salivary flow test (SSF) was performed. Patients were asked to chew a gaze swab for 2 min. A SSF value ≤ 2g per 2 min was considered as decreased saliva production (18).

Both tests were conducted at normal room temperature and participants were asked not to eat/drink/smoke for at least 2 h prior to the testing.

#### Clinical Assessments

ESSDAI (EULAR Sjögren's Syndrome disease activity index) (19) and EGA (Evaluator Global Assessment) (numeric rating scale from 0 to 10) were obtained to assess disease activity. At this point, the physician assessing disease activity was blinded to the results of objective measurement of dryness and PSS-QoL. In addition, free light chains (FLC)-κ (mg/L, normal range 3.30–19.40), FLC-λ (mg/L, normal range 5.71–26.30), Immunglobulin-G (IgG) (g/L, normal range 7.0–16.0), complement-factor 4 (C4) (g/L, range 0.100–0.400) and rheumatoid factor (RF) IgA (U/ml, normal range 0–20) were measured.

### Subjective Dryness Assessment

Patients' perspective of dryness was assessed by PSS-QoL (12), ESSPRI (11), XI (Xerostomia Inventory) (20), OSDI (Ocular Surface Disease Index) (21) and VAS-Scale (Visual Analog Scale) for the extent of sicca symptoms on different body parts (eyes, mouth, nose, skin, vagina) (scale from 0 to 100 mm).

PSS-QoL is a questionnaire to assess health-related quality of life (HRQL) in PSS patients. The questionnaire consists of two main categories: physical (discomfort and dryness) and psychosocial. The dryness-part asks for presence of dryness of the mouth, eyes, nose, skin, vagina and their accompanying dryness-symptoms and dryness-related consequences (12). For the purpose of this study, we calculated the variables PSS-QoL-dryness_mouth_ and PSS-QoL-dryness_eyes_ sum of all answers in this category (extent of dryness and dryness-related symptoms). PSS-QoL can be used in addition of VAS-scale to learn more about dryness-related symptoms as well as the disease-specific HRQL. While VAS-scales are unidimensional,PSS-QoL is a multidimensional tool.

ESSPRI evaluates the three main PSS domains with three questions about dryness, fatigue and pain. The items are measured on a numerical scale ranging from 0 to 10 (11). We used the question of ESSPRI-dryness for this study purpose.

Symptom severity of dry eyes symptoms was assessed by OSDI (21) and intensity of dry mouth by XI (20).

### Presentation of Perceived Dryness

Patients were categorized into groups based on the presence of subjective and/or objective dryness: (1) objective dryness only, (2) subjective dryness only, and (3) both, subjective and objective dryness. These groups were built separately for ESSPRI-dryness, PSS-QoL-dryness_mouth_, and PSS-QoL-dryness_eyes_. Perceived dryness was rated positive when ESSPRI-dryness was ≥1, PSS-QoL-dryness_mouth_ was ≥1, or PSS-QoL-dryness_eyes_ was ≥1. Objective dryness was rated positive when Schirmer's test, SSF, or USF were abnormal in the ESSPRI groups. In the PSS-QoL-dryness_mouth_ groups only results of SSF or/and USF were considered while Schirmer's test was considered in the PSS-QoL-dryness_eyes_ groups.

### Statistical Analysis

Statistical analyses were performed using IBM SPSS software (V25.0). Descriptive statistics were used to summarize the data. Quantitative results were compared dependent on the normal distribution by using the *t*-test for independent variables or Mann-Whitney U test.

We conducted the Spearman correlation coefficient to screen for correlation of objective/subjective dryness assessments and clinical variables. The *p*-values were not adjusted for multiple testing.

## Results

Patients' characteristics and clinical values of our PSS-cohort (*n* = 123) are depicted in Table 1.

**Table 1:** Patient characteristics and clinical variables.

**Demographics and clinical variables (*****n*** **=** **123)**	
Sex, female	91.9 (113)
Disease duration (years)	6.2 ± 5.3
Age (years)	60.1 ± 12.3
ESSDAI	3 [0–15]
ESSDAI activity level	
Low activity (<5)	81.0 (98)
Moderate/high activity(≥5)	19.0 (23)
ESSPRI	4.3 [0.3–9.7]
ESSPRI	
PASS (<5)	55.3 (68)
Non-PASS(≥5)	44.7 (55)
Schirmer's test, mm/5min	1.5 [0–35]
SSF, g/2min	1.5 [0–4.5]
USF, ml/5min	0.2 [0–5]
FLC-κ, mg/L	20.8 [8.7–163]
FLC-λ, mg/L	18.5 [7.0–124]
IgG, mg/L	14.8 [6.5–37.2]
PSS-QoL	34 [2–72.0]
PSS-QoL_psychosocial_	18 [0–43]
PSS-QoL_physical_	15 [0–32]
OSDI	54.2 [12.5–97.7]
ANA positive (≥1:80)	80.5 (91)
Ro positive	82.6 (95)
La positive	55.7 (64)
RF-IgA, positive	61.3 (68)
Corticosteroids	5.7 (7)
Pilocarpine	44.7 (55)
Immunosuppressants (Hydroxychloroquine, mycophenolate mofetil or others)	31.7 (39)

*Results are expressed as median [range], mean ± standard deviation or percent (number)*.

*ANA, antinuclear antibody; ESSDAI, EULAR Sjögren syndrome disease activity index; ESSPRI; FLC-κ, free light chain kappa; FLC-λ, free light chain lambda; IgG, immunoglobulin-G; La, anti-SSB/LA antibody; OSDI, ocular surface disease index; PASS, patient acceptable symptom state; PSS-QoL, primary Sjögren syndrome quality of life questionnaire; RF-IgA, rheumatoid factor—IgA; Ro, anti-SSA/RO antibody; SSF, stimulated salivary flow; USF, unstimulated salivary flow*.

The majority of patients were female (91.9%) with a mean disease duration of 6.2 years. Thirty percent (*n* = 37) had an additional disease of the thyroid, 4.1% had a MALT-lymphoma and 10.6% had a history of cancer (other than lymphoma).

Ophtalmologic assessments were performed in a subset of patients (*n* = 43).

### Measurement of Subjective and Objective Dryness

Subjective oral dryness was correlated stronger with objective measurements than ocular dryness (Table 2). PSS-QoL-Dryness_mouth_ revealed a moderate, negative correlation with SSF (corr_coeff_ = −0.409, *p* < 0.01) and USF (corr_coeff_ = −0.350, *p* < 0.01). Additionally, VAS-sicca scores of eyes and mouth showed moderately correlations with objective dryness measures (USF, SSF, Schirmer's test) (Table 2). The negative correlations indicate that a greater dryness burden was associated with lower tear and saliva production. Paradoxically, VAS-sicca-_eyes_ correlated with F-BUT (corr_coeff_ = 0.320, *p* < 0.05) and CSF (corr_coeff_ = −0.312, *p* < 0.05). In contrast, we found no significant correlation between ESSPRI, ESSPRI-dryness and any objective dryness test. The overall relationship between objective ocular and oral assessment methods are depicted in Supplementary Table 1. Objective dryness measures correlated moderate significantly. Pain and Fatigue (measured by ESSPRI) revealed a moderate correlation with subjective dryness measurement (PSS-QoL and Sicca VAS Scores). Additionally, we determined the relationship between PSS-QoL and Sicca-VAS scores as well as ESSPRI. PSS-QoL correlated strongly with ESSPRI (corr_coeff_ = 0.729, *p* < 0.01) and PSS-QoL_dryness_ correlated moderately with ESSPRI-dryness and VAS-sicca scores (for details see Supplementary Table 2).

**Table 2:** Correlation between objective and subjective dryness measurements.

	**SSF**	**USF**	**Schirmer's**
VAS-sicca_eyes_	ns	ns	ns
VAS-sicca_mouth_	−0.397^**^	−0.255^*^	ns
OSDI	−0.532^**^	−0.431^*^	−0.335^*^
XI	−0.502^**^	−0.390^**^	ns
PSS-QoL-Dryness_−MOUTH_	−0.409^**^	−0.350^**^	ns
PSS-QoL-Dryness-_EYES_	−0.316^**^	ns	−0.312^*^
PSS-QoL	ns	ns	ns
ESSPRI_dryness_	ns	ns	ns
ESSPRI	ns	ns	ns

** *p < 0.01*.

* *p < 0.05*.

*ns-not statistically significant*.

*ESSPRI, EULAR Sjögren Syndrome Patient Reported Index; OSDI, Ocular Surface Disease Index; PSS-QoL, Primary Sjögren Syndrome Quality of Life Questionnaire; SSF, stimulated salivary flow; USF, unstimulated salivary flow; VAS, Visual Analog Scale; XI, Xerostomia Index*.

### Dryness and Clinical Parameters

**Figure 1:**
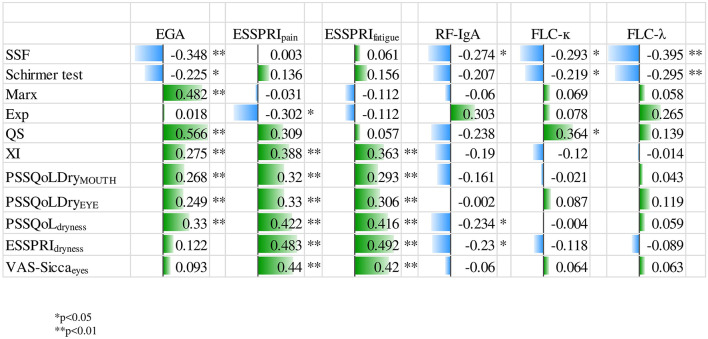
Relationship between dryness and clinical parameters. EGA, evaluators global assessment; ESSPRI, EULAR Sjögren syndrome patient reported index; Exp, expressibility; FLC-κ, free light chain kappa; FLC-λ, free light chain lambda; Marx, Marx line; PSS-QoL, primary sjögren syndrome quality of life questionnaire; QS, quality of meibomian gland secretion; RF-IgA, rheumatoid factor immunoglobin-A; SSF, stimulated salivary flow; VAS, visual analog scale; XI, xerostomia index.

The relationship between clinical parameters and dryness assessments (subjective and objective) is depicted in Figure 1. EGA revealed a moderate correlation with PSS-QoL_dryness_ (corr_coeff_ = 0.330, *p* < 0.01), VAS-siccamouth (corr_coeff_ = 0.195, *p* < 0.05) marx line (corr_coeff_ = 0.482, *p* < 0.01), as well as quality of meibomian gland secretion (corr_coeff_ = 0.566, *p* < 0.01). We did not identify any significant correlation between ESSDAI and dryness parameters. In addition, OSDI did not correlate with clinical parameters. Pain and Fatigue (measured by ESSPRI) revealed a moderate, significant correlation with subjective dryness measurement. The amount of serum IgG was significantly associated with SSF (corr_coeff_ = −0.218, *p* < 0.05). Interestingly, FLC-λ and FLC-κ showed an association with SSF and Schirmer's test (Supplementary Figure 1). The negative relationship between the parameters indicated that greater dryness burden was associated with lower immunological activity. This result lead us to build groups of different immunological activity (normal/high). Clinical parameters were compared within these groups. Table 3 demonstrates the group comparisons of normal/high RF-IgA, FLC-λ, FLC-κ and IgG with clinical parameters. Patients with immunological disease activity had higher clinical disease activity (ESSDAI, EGA), but lower subjective disease burden. Aspects of the patients' perspective like HRQL, fatigue and subjective dryness were higher in patients with normal immunological status according to RF-IgA and IgG. USF, disease duration, XI and OSDI did not show any significant differences between the groups (data not shown).

**Table 3:** Differences of clinical parameters in immunological groups.

	**Rheumatoid factor-IgA**			**Free-light chain** **κ**			**Free-light chain** **λ**			**IgG**		
	**Negative**	**Positive**	***p***	**Normal**	**High**	***p***	**Normal**	**High**	***p***	**Normal**	**High**	***p***
	**(0–20U/ml)**	**(>20U/ml)**		**(3.3–19.4 mg/L)**	**(>19.4 mg/L)**		**(5.7–26.3 mg/L)**	**(>26.3 mg/L)**		**(7.0–16.0 mg/L)**	**(>16.0 mg/L)**	
Schirmer's test	3 [0–35]	1.00 [0–35]	>0.05	**5 [0**–**35]**	**0 [0**–**35]**	***<0.01***	**3 [0**–**35]**	**0 [0**–**12]**	***<0.01***	1 [0–35]	1.5 [0–35]	>0.05
SSF	**2 [0.5**–**4.5]**	**1.4 [0**–**4.5]**	**<0.01**	**1.7 [0.2**–**4.5]**	**1.4 [0**–**3.5]**	**<0.05**	**1.5 [0**–**4.5]**	**0.7 [0**–**2]**	**<0.01**	**1.5 [0**–**4.5]**	**1 [0**–**3.5]**	**<0.05**
EGA	2 [0–5]	2 [0–7]	>0.05	2 [0–5]	2 [0–7]	>0.05	**2 [0**–**5]**	**4 [0**–**7]**	***<0.01***	**2 [0**–**5]**	**2 [1**–**5]**	***<0.01***
ESSDAI	***1 [0***–***15]***	***4 [0***–***11]***	***<0.01***	**1 [0**–**7]**	**4 [0**–**15]**	***<0.01***	**2 [0**–**15]**	**6 [0**–**14]**	***<0.01***	**1 [0**–**10]**	**5.5 [0**–**15]**	***<0.01***
ESSPRI_dryness_	***7 [1***–***10]***	***5 [0***–***10]***	***<0.01***	6.5 [1–7]	5.5 [0–10]	>0.05	6 [0–10]	5.5 [1–10]	>0.05	7 [1–10]	5 [0–10]	>0.05
ESSPRI_pain_	***4 [0***–***9]***	***3 [0***–***10]***	***<0.05***	3 [0–9]	4 [0–9]	>0.05	4 [0–9]	2.5 [0–8]	>0.05	**4 [0**–**9]**	**2.5 [0**–**10]**	***<0.05***
ESSPRI_fatigue_	***6 [0***–***10]***	***3.5 [0***–***10]***	***<0.01***	5 [0–10]	4 [0–10]	>0.05	5 [0–10]	3 [0–10]	>0.05	**5 [0**–**10]**	**3 [0**–**10]**	***<0.05***
ESSPRI	***5.7 [1.3***–***9.3]***	***3.8 [0***–***9.7]***	***<0.01***	9.67 [4.67–2]	4.5 [0–9]	>0.05	4.67 [0–9.33]	3.33 [1–9]	>0.05	**5.17 [1.33**–**9.33]**	**3.67 [0**–**9.67]**	***<0.05***
PSS-QoL_dryness_	***11 [3***–***19]***	***9 [0***–***22]***	***<0.05***	10 [1–19]	10 [1–22]	>0.05	10 [1–22]	10.5 [3–19]	>0.05	10 [1–22]	8 [0–19]	>0.05
AGE	***69 [37***–***88]***	***59.5 [26***–***80]***	***<0.01***	61.5 [35–84]	62 [33–88]	>0.05	60.5 [35–88]	66 [33–76]	>0.05	**64.5 [35**–**88]**	**56.5 [34**–**76]**	***<0.01***
PSS-QoL	***42.0 [11***–***72]***	***29.5 [2***–***68]***	***<0.01***	36 [11–72]	33 [8–69]	>0.05	35 [8–72]	32.5 [14–69]	>0.05	**40 [11**–**72]**	**28.5 [8**–**69]**	***<0.01***

*Results are expressed as median [range]*.

*EGA, evaluators global assessment; ESSDAI, EULAR Sjögren syndrome disease activity index; ESSPRI, EULAR Sjögren syndrome patient reported index; FLC-κ, free light chain kappa; FLC-λ, free light chain lambda; PSS-QoL, primary Sjögren syndrome quality of life questionnaire; SSF, stimulated salivary flow; VAS, visual analog scale*.

*Bold character is used to highlight the results with statistical significance*.

### Perceived Dryness

We categorized patients whether they perceived dryness and/or had reduced salivary or lacrimal flow. Analyzing perceived dryness according to ESSPRI revealed that 90% of patients were categorized into group 3 (both, perceived and objective dryness) while only a minority was categorized into groups 1 and 2. We could not further analyze these groups due to lack of statistical power.

Dryness recorded by the PSS-QoL (*n* = 100) identified patients with subjective dryness-symptoms of the eyes (17%) or mouth (25%) only (group 2) and patients who had both, subjective and objective signs of dryness (group 3, mouth 69%, eyes 71%). Patients exhibiting objective signs of dryness without the respective perception (group 1) were rare. Therefore, group comparisons were performed between group 2 and 3 only (Table 4). Patients with objective signs and subjective symptoms of dryness had higher clinical and immunological disease activity compared to patients with subjective symptoms only.

**Table 4:** Comparison of perceived dryness-groups with clinical parameters.

	**PSS-QoL Mouth**		***p***	**PSS-QoL Eyes**		***p***
	**subj**	**obj/subj**		**subj**	**obj/subj**	
EGA	**1.0 [0**–**5]**	**2.0 [0**–**7]**	**<0.05**	**1.0 [0**–**3]**	**2.0 [0**–**7]**	**<0.05**
ESSDAI	**1.0 [0**–**14**	**4.0 [0**–**15**	**<0.05**	3.0 [0–7	4.0 [0–15	ns
ESSPRI-pain	3.0 [0–9]	3.0 [0–10]	ns	**4.0 [1**–**8]**	**3.0 [0**–**10]**	**<0.05**
RF-IgM	19.0 [5–524]	31.0 [7–417]	ns	**10.0 [5**–**33]**	**26.0 [7**–**524]**	**<0.01**
FLC-λ	**15.1 [7.0**–**68.3]**	**20.1 [2.6**–**124.0]**	**<0.01**	**13.4 [7.0**–**54.5]**	**18.7 [2.6**–**124.0]**	**<0.01**
IgG	11.9 [6.5–37.2]	16.1 [7.4–33.8]	ns	12.8 [6.5–20.6]	14.8[7.4–37.2]	ns
C4	**0.2 [0.01**–**0.41]**	**0.17 [0.00**–**0.43]**	**<0.01**	0.2 [0.09–0.41]	0.18 [0.00–0.43]	ns
RF-IgA	**11.0 [1**–**500]**	**103 [0**–**500]**	**<0.01**	**4.5 [1**–**465]**	**63 [0**–**500]**	**<0.01**

*C4, Complementfactor 4; EGA, evaluators global assessment; ESSDAI, EULAR Sjögren syndrome disease activity index; ESSPRI, EULAR Sjögren syndrome patient reported index; FLC-λ, free light chain Lambda; IgG, Immunoglobulin G; obj/subj, objective and subjective dryness-group; PSS-QoL, primary Sjögren syndrome quality of life questionnaire; RF-IgA, rheumatoid factor immunoglobulin A; RF-IgM, rheumatoid factor Immunoglobulin M; subj, subjective dryness-group*.

*Bold character is used to highlight the results with statistical significance*.

## Discussion

In the present study, PSS-QoL-dryness correlated negatively with Schirmer's test and SSF. In contrast, we found no significant correlation between ESSPRI-dryness and any objective dryness test. Lower PSS-QoL-dryness was associated with higher immunological activity determined by increased levels of IgG, FLC and RF-IgA. Whereas patients with subjective signs of dryness only had lower immunological activity.

Dryness and dryness-related symptoms are the most important predictors for impairment of HRQL in PSS patients (2). However, assessment of the individual perception of dryness is difficult due to the existence of different psychologic constructs in symptom perception. For example, depression, fatigue or anxiety can cause or worsen dryness. Therefore, this complex construct should be evaluated as detailed as possible (22). Both, XI and OSDI correlated moderately with objective dryness tests showing a slightly stronger correlation than PSS-QoL. This finding is not surprising given the extensive and detailed assessment of specific symptoms in both tools. In contrast the PSS-QoL is a disease-specific questionnaire designed to evaluate HRQL. In addition to sicca symptoms, it also incorporates additional information on the impairments in daily living. To enhance feasibility and to ensure patient compliance a smaller but well-balanced set of questions was chosen to investigate the impact of sicca on HRQL. An overall VAS like ESSPRI-dryness might not cover the real estimate of dryness. ESSPRI-dryness did not correlate with salivary or lacrimal flow, which is in line with a prior study only finding a weak agreement with these objective tests (9). In contrast, ESSPRI correlated well with the other ESSPRI VAS scores, —pain and fatigue. This may imply that ESSPRI-dryness reflects a more general concept of dryness influenced by pain and fatigue. When we asked to rate more specific concepts of dryness by using separate VAS scores for each affected location, we could find a correlation to the results of objective tests. This correlation was even stronger for the PSS-Qol, which asks for specific symptoms of dryness rather than using a VAS score in its dryness domains. Nevertheless, clinical trials are using ESSPRI to evaluate a possible improvement of dryness (23). It remains unknown if clinical trials in PSS may have shown improvement in dryness if more specific concepts had been assessed. Similar to dryness, fatigue is a complex construct. However, significant improvement could be detected when using a detailed assessment tool: In a recent phase-II study an anti-BAFF antibody (Ianalumab) induced significant improvement in domains of the MFI (Multidimensional Fatigue Inventory) including domains of general fatigue, physical fatigue and reduced activity (23). In the same trial, no significant improvement of the overall ESSPRI or ESSPRI-fatigue was reported. Similarly, the evaluation of different domains and symptoms of dryness could be a better approach to evaluate the patient perspective in future trials.

In line with a previous report, perceived oral dryness correlated stronger with objective dryness assessments than ocular dryness (9). A possible reason may be the reduced corneal sensation in advanced ocular surface damage (6). In line with this hypothesis, we observed a lower VAS-sicca_eyes_ in patients with increased corneal fluorescein staining score and reduced fluorescein break-up time.

We observed a lower perception of sicca symptoms in patients with increased serum IgG and RF-IgA suggesting immunological active disease. Interestingly, high immunological activity and higher clinical disease activity measured by ESSDAI were associated with a lower subjective disease burden such as extent of dryness and impact on HRQL (24). Intriguingly, serum FLC-λ/κ were associated with objective ocular and oral dryness tests. FLC-λ is associated with ocular signs and symptoms (25). In general, FLC-λ/κ are of growing interest for evaluation of treatment response and monitoring of disease activity, while they are usable as biomarker for B cell activity. Elevated FLC-λ/κ are indicative for MALT lymphoma and associated with systemic disease activity (24).

PSS can manifest in a variety of symptoms. Therefore, different approaches to stratify PSS patients into different groups have been performed: Patients were divided into groups of predominantly glandular symptoms and those with extra-glandular manifestations (26). A further stratification of PSS patients resulted in four major groups: (1) low symptom burden, (2) high symptom burden, (3) dryness dominant with fatigue and (4) pain dominant with fatigue (10). Our approach considers objective and subjective aspects of dryness: patients with objective and subjective signs of dryness had more clinical and immunological disease activity, while patients with subjective dryness signs only had a higher subjective disease burden, more pain and impaired health-related quality of life. In this context, it should be pointed out that immunological activity has been successfully targeted in different clinical trials in PSS without improvement of the patient perspective (24, 27).

The strengths of this study include our well-characterized PSS-cohort, that allowed us to include various different parameters into the analysis. However, there are limitations: First, ocular parameters like marx-line or lissamine-green staining were only available from half of the patients. Nevertheless, data of the Schirmer's test were available of 79% of the patients. Furthermore, correlations and the results of patient stratification should be validated in a larger cohort. Further study will validate our results and provide long-time followed-up data.

In summary, we identified two PSS patient-groups: (1) patients with high perceived dryness and impaired quality of life and (2) patients with lower perception of dryness but higher clinical and immunological disease activity. Although widely used, the ESSPRI might not allow a precise evaluation of the patient perspective of dryness. PSS-QoL allows evaluating dryness of all affected body regions besides other aspects of HRQL and is a useful tool to measure the perception of dryness.

## Data Availability Statement

The raw data supporting the conclusions of this article will be made available by the authors, without undue reservation.

## Ethics Statement

The studies involving human participants were reviewed and approved by institutional review board of the Medical University of Graz. The patients/participants provided their written informed consent to participate in this study.

## Author Contributions

All authors participated in the development of the project, the interpretation of the data, the manuscript preparation, and approved the current version of the manuscript.

## Conflict of Interest

The authors declare that the research was conducted in the absence of any commercial or financial relationships that could be construed as a potential conflict of interest.
