# Supplementary material for: Go Ask Your Patients! PSS-QoL Reported Perception of Dryness Correlates With Lacrimal and Salivary Flow in Primary Sjögren's Syndrome
Source: Front Med (Lausanne). 2021 Apr 15;8:660580. doi: 10.3389/fmed.2021.660580 (PMC8081854; doi:10.3389/fmed.2021.660580)
Supplement: Supplementary file 1 [file Data_Sheet_1.docx]

Supplemental table 1: Relationship between objective dryness measurements

|  | SSF | USF | Schirmer’s | F-BUT | LG | CFS | Marx | Exp |
| --- | --- | --- | --- | --- | --- | --- | --- | --- |
| SSF |  |  |  |  |  |  |  |  |
| USF | 0.508^**^ |  |  |  |  |  |  |  |
| Schirmer’s | ns | 0.465^*^ |  |  |  |  |  |  |
| F-BUT | ns | ns | 0.353^*^ |  |  |  |  |  |
| LG | ns | ns | ns | ns |  |  |  |  |
| CFS | ns | ns | ns | -0.360^*^ | 0.724^**^ |  |  |  |
| Marx | ns | ns | ns | ns | ns | ns |  |  |
| Exp | ns | ns | ns | ns | ns | ns | ns |  |
| QS | ns | ns | ns | ns | ns | ns | 0.468^**^ | ns |

**p<0.01

*p<0.05

ns-not statistically significant

CFS=corneal fluorescein staining; Exp=Expressibility; F-BUT= fluorescein tear film break-up time; LG=lissamine-green staining; Marx=Marx line; QS=Quality of meibomian gland secretion; SSF=stimulated salivary flow; USF=unstimulated salivary flow.

Supplemental table 2: Correlations between subjective dryness measurements

|  | PSS-QoL-Dryness_-MOUTH_ | PSS-QoL-Dryness-_EYES_ | PSSQoL_dryness_ | PSS-QoL |
| --- | --- | --- | --- | --- |
| ESSPRI_dryness_ | 0.441^**^ | 0.486^**^ | 0.520^**^ | 0.480^**^ |
| ESSPRI | 0.410^**^ | 0.448^**^ | 0.537^**^ | 0.729^**^ |
| VAS-sicca_mouth_ | 0.471^**^ | 0.386^**^ | 0.463^**^ | 0.369^**^ |
| VAS-sicca_eyes_ | 0.323^**^ | 0.521^**^ | 0.441^**^ | 0.359^**^ |

**p<0.01

ESSPRI=EULAR Sjögren Syndrome Patient Reported Index; PSS-QoL=Primary Sjögren Syndrome Quality of Life Questionnaire; VAS=Visual Analogue Scale.

Supplemental figure 1*:* Correlation of free-light chains and objective dryness measurement


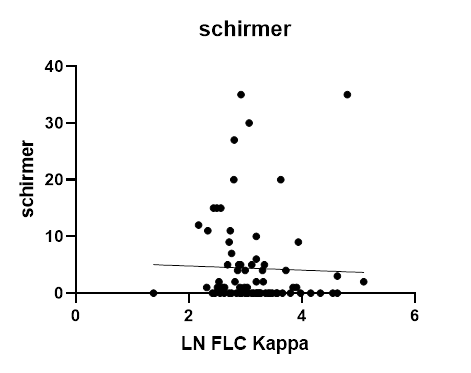

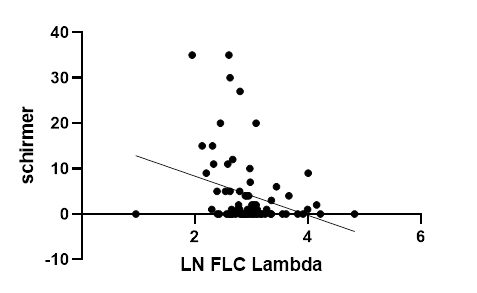

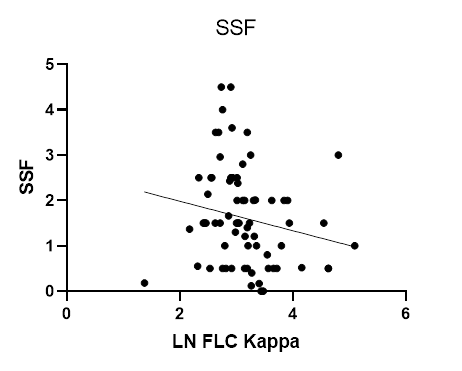

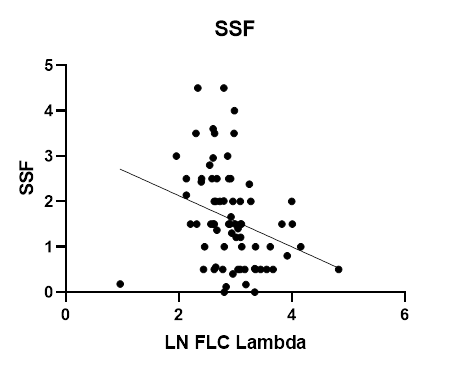


r= -0.293

(p<0.05)

r= -0.219

(p<0.05)

r= -0.295

(p<0.01)

r= -0.395

(p<0.01)
